# The Value of *H3K27me3* Immunohistochemistry in Differentiating Malignant Peripheral Nerve Sheath Tumour with Its Histologic Mimickers

**DOI:** 10.31557/APJCP.2020.21.3.699

**Published:** 2020-03

**Authors:** Nurulhasanah Mustapar, Muhamad Syahrul Fitri Zawawi, Sharifah Emilia Tuan Sharif

**Affiliations:** 1 *Department of Pathology, *; 3 *Department of Orthopedics, School of Medical Sciences, Universiti Sains Malaysia, Health Campus, *; 4 *Department of Pathology, Hospital Universiti Sains Malaysia, Kubang Kerian, Kelantan, *; 2 *Department of Pathology and Laboratory Medicine, International Islamic University Malaysia, Kuantan, Pahang, Malaysia. *

**Keywords:** H3K27me3, IHC, Malignant peripheral nerve sheath tumour

## Abstract

**Background:**

Diagnosis of malignant peripheral nerve sheath tumor (MPNST) is rather challenging due to its divergent morphologic heterogeneity and lack of specific ancillary test. The emergence of H3K27 trimethylation (*H3K27me3*) as a new immunohistochemistry (IHC) marker for MPNST have recently available to assist pathologists in differentiating MPNST from other histologic mimics. We aim to study the expression pattern of *H3K27me3* in MPNST and its histologic mimickers and their association with the clinicopathological data.

**Methodology:**

A total of 59 benign and malignant spindle cell tumours (18 MPNST and 41 of its histologic mimickers which included 10 schwannoma, 13 neurofibroma, 4 synovial sarcoma, 3 fibrosarcoma, 2 gastrointestinal stromal tumour (GIST), 4 leiomyosarcoma, 1 spindle cell liposarcoma, 1 solitary fibrous tumour, 2 low grade fibromyxoid sarcoma and 1 unclassified spindle cell sarcoma), diagnosed from January 1998 to April 2018 in Hospital Universiti Sains Malaysia (HUSM) were tested for *H3K27me3* by IHC. The MPNST histological grade was assessed based on the French Fe’de’ ration Nationale des Centres de LutteContre le Cancer (FNCLCC) for 3 tiers system (low grade, intermediate grade and high grade). The clinicopathological data were retrieved from the patients’ record.

**Results:**

A total of 61.1% (11/18 MPNST) showed loss of *H3K27me3* expression which is statistically significant as compared to its histologic mimics (p<0.001). Similar findings (p=0.026) were also observed in high grade MPNST (81.8%), intermediate grade MPNST (100%) and 0% in low grade MPNST.

**Conclusion:**

*H3K27me3*, combined with other panel of markers, is useful in MPNST diagnosis to differentiate it from the histological mimickers.

## Introduction

Malignant peripheral nerve sheath tumour (MPNST) is particularly rare with an incidence of 0.001% (Perrin and Guha, 2004) in the general population and representing approximately 4% of all soft tissue sarcomas (Toro et al., 2006). This soft tissue neoplasm has gained an interest due to its diversity of histologic appearances, overlapping pattern, the rarity of the tumour, and the need of supportive and confirmatory tests which often renders MPNST as diagnostically challenging neoplasm. All these may justify to the delay in diagnosis. MPNST is rare, aggressive soft tissue sarcomas associated with poor prognosis. 

The diagnosis of MPNST relies on morphology and IHC interpretation. Differentiating MPNST from other types of spindle cell neoplasm is important for prognosis and management. Several markers have been suggested to distinguish MPNST from other soft tissue tumours. These include S-100 protein, SOX10, epithelial membrane antigen (EMA), cytokeratins, CD34, HMB45 and desmin (Weiss, 2008). None of these markers are specific for MPNSTs and mostly used to narrow down the diagnosis of spindle cell tumour. The emergence of H3K27 trimethylation (*H3K27me3*) as a new immunohistochemistry marker for MPNST have recently available to assist pathologists in differentiating MPNST from other histologic mimics. However, it also remains conflicting results for H3K27me3. Prieto-Granada et al., (2016) found that *H3K27me3 *immunohistochemistry has good sensitivity and robust specificity for the diagnosis of MPNST, while others noted loss of expression in other type of sarcomas too, which included radiation-associated angiosarcomas (Mentzel and Kiss, 2018) and dedifferentiated chondrosarcoma (Makise et al., 2019). In this study we investigated the expression of *H3K27me3* in differentiating MPNST from other types of spindle cell tumour and its association with its histological grading and clinicopathological data.

## Materials and Methods

A total of 59 cases was included in this study, consisted of 18 cases diagnosed histologically as MPNST and 41 cases of other benign and malignant spindle cell neoplasm as control group and labelled as mimickers, retrieved from registry book and Pathology computerized registry data files (from 1998 until 2018). The clinicopathological data for all cases which included age, clinical presentation, site and depth of tumour involvement, tumour size, type of sample, patients’progress such as presence of local recurrence and metastasis after diagnosis, were also obtained from patients’ record notes. The study obtained its ethical clearance from the Ethical Committee, Universiti Sains Malaysia (JEPEM Code: USM/JEPeM/17020148).

*Histological assessment*

The tissue slides were deparaffinized and hydrated using standard procedures and stained with hematoxylin and eosin (H&E). The slides were viewed using Olympus CX31 light microscopy to assess the morphology of tumour and grading of MPNST. The grading system is based on tumour differentiation, mitotic count and tumour necrosis in FNCLCC grading system. A 3-tiered grading system with low-grade (LG), intermediate grade (IG) and high-grade (HG) MPNSTs is used, with the LG end being characterized by neurofibroma (NF)-like tumors composed of a cellular proliferation of bland spindle cells that appear to be in a continuum with the so-called atypical/cellular neurofibroma. The typical morphologic picture defining a conventional HG MPNST is represented by a fascicular, monomorphic spindle cell proliferation with a “marbled” low-power appearance and areas of geographic necrosis. Finally, examples exhibiting histomorphologic features that deviated from these classic LG and HG patterns were classified under intermediate MPNST (Prieto-Granada et al., 2016).

*Immunohistochemical (IHC) staining for H3K27 trimethylation*

IHC staining was performed to the paraffin-embedded tissue blocks according to standard procedures (Abcam Code Ab192985) using pressure cooker antigen retrieval (Tris-EDTA buffer solution pH 9.0) method. The monoclonal primary antibody *H3K27me3 *at 1:200 was applied to the sections. Sections of mimickers without primary antibodies served as negative controls, and sections of mimickers stained with primary antibodies for each antibody served as positive controls. All these were run together with each batch of staining. Tissue from colon was used as positive control for *H3K27me3* staining.

Its expression was quantified in the various samples examined using a scoring method adopted from Cleven et al., (2016). A mean percentage of nuclear staining was determined at a magnification of 400x, and scored 0 (0 cell positive), 1+ (1-24% of cell positive), 2+ (25-49% of cell positive) and 3+ (>50% of cell positive). The 0 score is considered loss of expression. Microscopy for immunoreactivity was evaluated by a single independent pathologist who is blinded to the case. Data were statistically analyzed by IBM SPSS version 24, using chi-square test or fisher-exact test. 

## Results

*Epidemiological and clinicopathological data*

A total of 59 participants involved in the present study. A total of 18 MPNST cases and 41 of its histologic mimickers (10 schwannoma, 13 neurofibroma), 4 synovial sarcoma, 3 fibrosarcoma, 2 gastrointestinal stromal tumour (GIST), 4 leiomyosarcoma, 1 spindle cell liposarcoma, 1 solitary fibrous tumour, 2 low grade fibromyxoid sarcoma and 1 unclassified spindle cell sarcoma) were included in this study. The result of the data showed that the mean age of the participants is 40.76 years old (SD=17.21). Our study shows a bimodal distribution of MPNST patients which were most prevalence in the 12-year-old and younger age group and at 40-59 year old age group with mean age of diagnosis was 32.83 (SD=20.54). Ten out of 18 (55.6%) of MPNST cases were sporadic and 44.4% (8 out of 18) were NF-1 associated MPNST.

Morphologically 14 cases (77.8%) were classical spindled type, 3 cases (16.7%) were malignant triton tumour and 1 case (5.6%) was epithelioid MPNST. For histologic grades, 77.8% (11/18) of MPNST cases were high grade, 11.1% (2/18) were intermediate grade and low grade cases respectively. 

In term of patients’ outcomes, 7 out of 18 MPNST cases (38.9%) appeared with no local recurrence or distant metastases. Six out of 18 (33.3%) cases noted to have distant tumour metastasis at diagnosis and less than 5 years after diagnosis. Lungs, intraspinal and bone were the predominant sites of metastases. Three out of 18 cases (16.7%) show disease recurrence and 2 out of 18 cases (11.1%) resulted in death less than 5 years after diagnosis.

*Pattern of expression of H3K27me3 in MPNST*

We evaluated the expression of *H3K27me3* in MPNST. Of the 18 tumours evaluated for *H3K27me3* expression, 14 are high grade MPNST and remaining 2 each are of intermediate grade and low grade MPNST. Majority of high grade MPNST (9/14) and all of the intermediate grade MPNST showed loss of *H3K27me3* expression. None of the low-grade cases show loss of *H3K27me3* expression. However, 1 case of high grade MPNST (5.6%) show strong immunoreactivity towards *H3K27me3* expression (Table 1). In our study, we also found that 75% of NF1-associated MPNST showed loss of *H3K27me3* expression as compared to only 50% sporadic MPNST showed negative nuclear immunoreactivity towards *H3K27me3* marker. However, our study found that this finding was not statistically significant (p=0.367).

*Pattern of expression of H3K27me3 in Mimickers*

Overall 38 cases of benign and malignant histologic mimickers, express *H3K27me3* (Table 3). In our study, among benign peripheral nerve sheath tumour, none of them show loss of *H3K27me3* expression. One out of 4 synovial sarcoma cases show loss of *H3K27me3 *expression. The other mimickers show heterogenous *H3K27me3* expression ranging from weak to strong nuclear immunoreactivity (1+ to 3+) (Table 2).

## Discussion

In general, the malignant peripheral nerve sheath tumour is typically seen in adult life because most tumour occur in patients 20 to 50 years of age with a median age of about 35 (Weiss, 2008). In our study, the median age was 28.5 years old ranged from the youngest age of 3 year old and the eldest was 81 year old. Although MPNST is most prevalent in adolescents and young adults, our study noted 4 cases (22.2%) of the MPNST cases were found in the 12 and younger age group. MPNST in childhood are recognized but rare and it may affect patient in a wide age range from birth to eighth decades. This was supported by Kudesia et al., (2014), in which the incidence of MPNST among paediatric patient, whom age less than 5 months old was only about 1.7%. In this study, we noted that among MPNST cases, 8 out of 18 patients has associated background history of neurofibromatosis 1 (NF1) with skin manifestation. Among patient with NF1, majority are diagnosed with MPNST at slightly earlier age with equal male to female distribution as compared to those with sporadic MPNST (Kudesia et al., 2014).

In our study, we found that the commonest site of tumour were upper extremities, followed by the trunk and upper extremities. This findings showed similarity with previous studies (Doorn et al., 1995; Ducatman et al., 1986; Kar et al., 2006; Prieto-Granada et al., 2016). They found this tumour occur predominantly in the extremities, followed by the trunk/retroperitoneum and head and neck region. This findings explain the fact that most MPNSTs arise in association with major nerve trunks, including the sciatic nerve, brachial plexus and sacral plexus. Besides, it has been described at various anatomical sites including intraspinal and various visceral (Aydin et al., 2004; Bees et al., 1997; Kóbori et al., 2008; Panigrahi et al., 2013).

A tumour can behave like benign, such as in the form of well circumscribed mass, superficial mass with indolent presentation and this may lead to delay in the patients’ management. This type of tumour is either asymptomatic or may cause minimal discomfort. Many patients reported pain and neurological deficits at time of presentation. In our study, this commonly slow enlarging mass may exhibits rapid growth and we found that all of MPNST cases in our study presented with lesions more than 5cm as compared to benign peripheral nerve sheath tumour which in majority of cases presented with tumour less than 5cm at time of diagnosis. In majority of cases the duration of the tumour are less than 1 year upon diagnosis. 

MPNSTs have a relatively poor prognosis in comparison with other soft-tissue malignancies. The five-year survival for adults and children varies from 35% to 50% (Arenas et al., 2016). Most MPNSTs are aggressive, high grade sarcomas with a high likelihood of local recurrence (40% to 65%), and distant metastases (40% to 68%.) (Arenas et al., 2016). From previous case series, they found that MPNST frequently metastasize to the lungs followed by bone whereas lymph node metastases are uncommon (Anghileri et al., 2006; Arenas et al., 2016; Ducatman et al., 1986; Fenzi et al., 1995). Other sites of metastasis include liver, brain, soft tissue, skin, and retroperitoneum. In our study, nine cases showed distant metastases and local recurrence with majority metastasize to the lung followed by other sites including intraspinal, liver and testes. However, the data from our study were limited because some of the patient had missing data or defaulted follow up. We had difficulty in tracing record of the patient and getting adequate information, especially those who were previously referred case and treated outside of our center. MPNSTs have poor outcome if untreated. Unfortunately, two of our patient developed lung metastases and succumbed to death after diagnosis with advanced MPNST in spite of a wide excision and additional chemotherapy. 

Although MPNST is rare in the paediatric age group, based on our study, diagnosis should be considered in children without NF1 with a rapidly evolving and painful mass in the distribution of a peripheral nerve. Early diagnosis and referral to multidisciplinary team are important in ensuring the best diagnosis and optimal therapy in this young age. In this study, we would like to emphasize on the important of this marker to help identify MPNST early in order for patient to get appropriate treatment earlier.

From our study, the predominant subtype was spindle cell type (77.8%). It is very crucial to differentiate it from other type of spindle cell sarcoma. The diagnosis of spindle MPNST is challenging especially if the mass arising from unusual site. In one of our paediatric cases, it was arising from a pelvic region and the morphological pattern was purely spindle. In this case, synovial sarcoma was one of the differentials and interestingly it showed moderate to strong nuclear immunoreactivity to TLE1 in which rendered more challenges to the pathologist. However, TLE-1 positivity can also be seen in about 15% of MPNST and about 8% in Solitary Fibrous Tumour (Foo et al., 2011). Here, we could see the role of H3k27me3 in which it showed loss of its expression thus, it confirmed the diagnosis of MPNST especially when the molecular test is not available in the centre to demonstrate the presence of translocation t(X;18) in synovial sarcoma.

**Figure 1:**
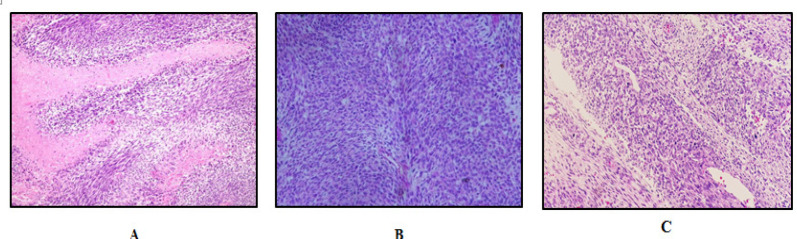
Histomorphology Features of MPNST. (A), A characteristic hypercellular alternating with hypocellular areas with extensive geographical necrosis in MPNST (H and E 200x); (B), Herring-bone pattern-like architecture may also mimics fibrosarcoma(H and E 200x); (C) Perivascular accentuation is a common feature in MPNST(H and E 200x)

There are several reported cases of a rare spindle MPNST arising in a peripheral nerve, in which the clinical and radiological findings were indistinguishable from benign peripheral nerve sheath tumour, lead to insufficient initial treatment. Based on morphological criteria including high cellularity, nuclear atypia and mitotic activity, pathologist try to differentiate MPNST from atypical neurofibroma. Microscopically the tumours were composed of highly cellular spindle-shaped cells arranged in hypercellular and hypocellular areas with perivascular hypercellularity, characteristic of MPNST (Figure 1).

MPNST is the malignant counterpart of peripheral nerve sheath tumour and the main differential diagnosis. Logically a greater degree of cellularity, pleomorphism and higher mitotic activity are criteria used to classify the lesion as malignant. A distinctive, rare subtype of MPNST that raises separate differential is characterized by a predominance of large epithelioid cells, such as epithelioid MPNST. These tumours are relatively more common in superficial sites and strongly express S100 protein. In our study only one epithelioid MPNST case encountered and similar to previous study, it expressed diffuse and strong S100 protein and showed weak immunoreactivity towards *H3K27me3* IHC. 

Although S100 protein is a marker of nerve sheath differentiation, it has limited diagnostic utility as MPNSTs are often negative or very focally positive, (50-90%). Small percentage of MPNST cases (2%-15%) were found to express TLE-1. However the positive expression were either weak (1+) or moderate (2+) staining (Foo et al., 2011). Therefore, in practice usually strong staining (3+) for TLE-1 argues strongly against a diagnosis of MPNST. 

Accurate diagnosis is crucial in choosing the correct treatment given. In this study we found one of the cases was diagnosed and treated as Ewing Sarcoma as initial biopsy of the case was reported as possible Ewing sarcoma. However, the final diagnosis from the excised tumour, it was malignant peripheral nerve sheath tumour. The discrepancy of the diagnoses showed the heterogeneity of the tumour itself which may resembled different morphological features in a small biopsy. Over the past two decades, a few studies have shown that diagnostic agreement in soft tissue neoplasm between primary institutional diagnosis and reviewer diagnosis ranges from 53% to 73% (Thway et al., 2014). Overall diagnostic discrepancies range from 28% to 35%, of which minor discrepancies constitute 7% to 16% and major discrepancies 11% to 25% (Thway et al., 2014). The rarity of soft tissue tumours and its constantly evolving histopathological criteria, poses a challenge for general pathologists resulting in referral to centres with a soft tissue pathologist. The implication of this is that an inappropriate panel of IHC stains would be performed, with or without IHC misinterpretation (BAO et al., 2017). Thus, additional diagnostic markers are needed to aid in this often challenging differential diagnosis (Cleven et al., 2016).

In a study on cancer development by epigenetic modifications, they have found that proteins of the polycomb group which are transcriptional repressors that modify chromatin, regulate cell fate, and promote cancer growth actually play an important role in pathogenesis of MPNST (Schaefer et al., 2016). In MPNST, inactivation of polycomb repressive complex II (PRC2) promotes cell proliferation and tumor growth. Inactivation of PRC2 is therefore expected to result in both loss of *H3K27me3* and altered DNA methylation (Rohrich et al., 2016).

**Figure 2:**
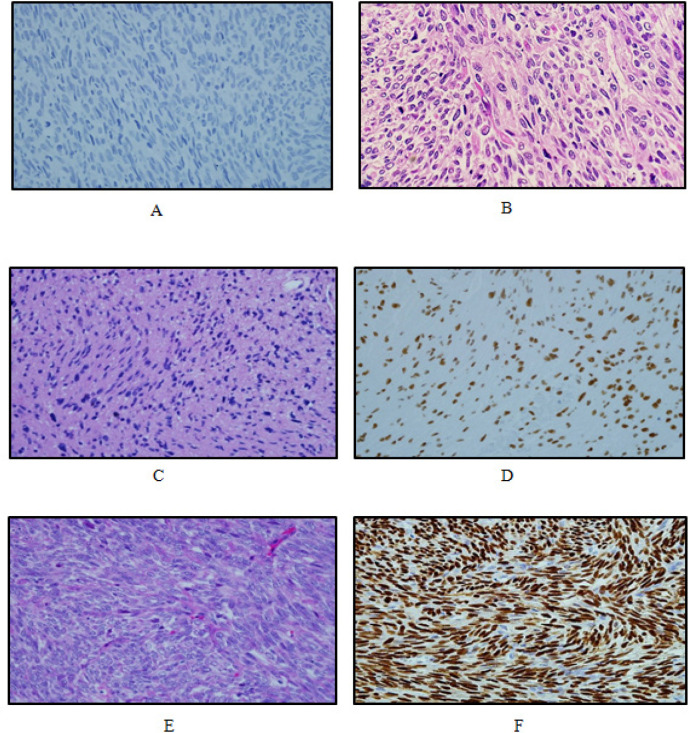
Variable *H3K27me3* Expression in MPNST Histologic Mimickers. (A), High grade MPNST exhibit loss of *H3K27me3* expression (IHC 400x); (B) Malignant triton tumour shows sheets of spindle cells with rhabdomyoblastic differentiation (H and E 400x). (C, E) Histologic mimickers -Schwannoma and Monophasic Synovial sarcoma (H and E,400x) and (D,F) positive expression of *H3K27me3* (IHC, 400x)

Many studies had shown that inactivation of the polycomb repressive complex 2 (PRC2), has recently been identified in 70–90% of malignant peripheral nerve sheath tumour (Lee et al., 2014; Prieto-Granada et al., 2016; Schaefer et al., 2016). The recent discovery of frequent inactivation of PRC2 in MPNSTs suggests that immunohistochemical detection of *H3K27me3* could be of diagnostic aid (Schaefer et al., 2016). Other study suggest that PRC2 inactivation in MPNST may occur during progression to higher grades (Schaefer et al., 2016). In our study, immunohistochemistry of *H3K27me3* proved to show loss of expression in 61.1% of MPNST, comparable to the findings of a previous study which showed 51% negative for *H3K27me3* (Schaefer et al., 2016), and 69% negative for* H3K27me3* of all MPNSTs (Prieto-Granada et al., (2016). It showed that MPNST group have the highest number of *H3K27me3* negative expression, while mimickers group mostly displayed strong immunoreactivity towards *H3K27me3* (Figure 2). Our findings are consistent with previous reports showing there were significant association between *H3K27 *expression and type of cases (p<0.001). These findings suggest that *H3K27me3 *loss is definitely a more specific marker for MPNST and useful in differential diagnosis, particularly for tumors that lack expression of supportive Schwann cell markers (examples, S100 protein, SOX10 and GFAP) and among high grade spindle cell sarcomas. 

Majority of MPNST showed loss of *H3K27me3 *(61.1%) whereas only 7.3% were loss in non-MPNSTs (histologic mimickers). The findings were also observed by Cleven et al., (2016), who found that 24 out of 341 (7%) cases of other spindle cell tumours were negative for H3K27me3. From this study, we found that the marker also played a role in differentiating benign peripheral nerve sheath tumour and malignant peripheral nerve sheath tumour. In our study, both neurofibroma and schwannoma show moderate to strong expression of *H3K27me3* with a p value of <0.001. This indicate that there is a significance difference in expression of *H3K27me3* between benign peripheral nerve sheath tumour and malignant peripheral nerve sheath tumour (TABLE 3). This is in line with the findings of a previous study that reported *H3K27me3 *as better marker in differentiating MPNST from benign peripheral nerve sheath tumour in particular neurofibroma and other morphological mimickers (Schaefer et al., 2016). In our study, 25% of synovial sarcoma (SS) and 33.3% of fibrosarcomatous dermatofibrosarcoma protuberans (DFSP) showed loss of H3K27 trimethylation expression. The 25% of SS cases warrant molecular test for diagnostic confirmation. On the other hand in another study by Cleven et al. (2016) on MPNST and other spindle cell tumours, loss of *H3K27me3 *was reported in 60% of synovial sarcomas and 38% of DFSP. Our results are almost in agreement with the above study in which loss of expression was also observed in non-MPNST. This highlight the possibility that this antibody is not specific to MPNST. 

However, the marker was significantly showed to be useful in differential diagnosis of MPNST when combine with other ancillary test (Prieto-Granada et al., 2016; Schaefer et al., 2016).

The association between *H3K27me3* expression and MPNST histologic grades is another important finding. In our study, loss of *H3K27me3* was observed in 64.3% of high grade, all of the intermediate grade and none of the low grade MPNST. Our study showed there was significant association between the histologic grades and *H3K27me3* expression. Most of the *H3K27me3*-negative MPNST (9/18) were high grade with 3 cases showed local recurrence, 4 cases showed distant metastases and 2 cases end up with death while none of the low grade or intermediate grade having any local recurrence or distant metastases. This is in keeping with previous findings where loss of positivity for *H3K27me3* is associated with poor prognosis in MPNST (Schaefer et al., 2016). In this study, there is no significant association between morphologic subtypes of MPNST and the expression of *H3K27me3 *immunoreactivity. This was supported by Prieto-Granada et al., (2016) where they found no significant differences in staining between different MPNST subtypes. However, all malignant triton tumour showed loss of expression and associated with poor prognosis and more aggressive behavior (Cleven et al., 2016). We also found that there was no significant difference in the expression of *H3K27me3* immunoreactivity between NF1 associated MPNST and sporadic MPNST.

Recent study by Prieto-Granada et al., (2016) showed loss of expression of *H3K27me3* in majority of sporadic MPNST cases as compared to NF1-associated MPNST and conclude that *H3K27me3* analysis has good sensitivity and robust specificity for the diagnosis of MPNST, particularly without clinical history of NF-1. Prieto-Granada et al., (2016) found that the sensitivity of *H3K27me3* for sporadic MPNST and radiation-induced MPNST are 94 % and 93%, respectively. However, further study needs to be carried out to determine the sensitivity and specificity of this marker for diagnosis of MPNST. For the clinical management of these patients, it may be of value to be able to identify MPNST among these sarcomas to select patients who might be candidates for novel targeted therapeutic approaches (Schaefer et al., 2016).

Since most of the panel of IHC in diagnosis MPNST are of limited value, our results had shown that *H3K27me3 *is a potentially good immunohistochemical diagnostic marker. Though we found it is not to be a specific marker, but it definitely will give an added value when use in combination with other IHC panels especially in differentiating MPNST from its histologic mimickers. 

**Table 1:** The Expression of *H3K27me3* ImmunohistoChemistry between Low Grade, Intermediate Grade and High Grade MPNST

Variable	Low Grade MPNST n (%)	Intermediate Grade MPNST n (%)	High Grade MPNST n (%)	P value^a^
H3K27 tri-methylation				0.026
0	0 (0.0)	2 (100.0)	9 (64.3)	
1+	0 (0.0)	0 (0.0)	4 (28.6)	
2+	2 (100.0)	0(0.0)	0 (0.0)	
3+	0 (0.0)	0 (0.0)	1 (7.1)	

^a^, Fisher exact test was applied

**Table 2:** The Expression of *H3K27me3* Immunohistochemistry in Histologic Mimickers

Variable	*H3K27* tri-methylation, n (%)			
	0	1+	2+	3+
Schwannoma	0 (0.0)	2 (20.0)	2 (20.0)	6 (60.0)
Neurofibroma	0 (0.0)	3 (23.1)	6 (46.2)	4 (30.8)
Synovial sarcoma	1 (25.0)	0 (0.0)	0 (0.0)	3 (75.0)
Fibrosarcoma	1 (33.3)	1 (33.3)	1 (33.3)	0 (0.0)
GIST	0 (0.0)	1 (50.0)	1 (50.0)	0 (0.0)
Leiomyosarcoma	0 (0.0)	1 (25.0)	1 (25.0)	2 (50.0)
Unclassified Spindle cell sarcoma	0 (0.0)	0 (0.0)	0 (0.0)	1 (100.0)
Solitary fibrous tumour	0 (0.0)	0 (0.0)	1 (100.0)	0 (0.0)
Spindle cell liposarcoma	0 (0.0)	0 (0.0)	1 (100.0)	0 (0.0)
Low grade fibromyxoid sarcoma	1 (50.0)	0 (0.0)	0 (0.0)	1 (50.0)

**Table 3:** The Expression of *H3K27me3* ImmunohistoChemistry between MPNST and Its Mimickers

*H3K27* tri-methylation	MPNST n (%)	Mimickers n (%)	P value^a^
0	11(61.1)	3(7.3)	<0.001
1+	4(22.2)	8(19.5)	
2+	2(11.1)	13(31.7)	
3+	1(5.6)	17(41.5)	

^a^, Fisher exact test was applied.
